# Hudson River Association of Dental Surgeons

**Published:** 1867-06

**Authors:** 


### HUDSON RIVER ASSOCIATION OF DENTAL SURGEONS.

This Association was formed at Poughkeepsie, April 25, 1867. There was a general attendance of members of the profession residing upon and contiguous to the Hudson River.

The following officers were elected:

*President.*—Dr. E. D. Fuller, Peakskill.

*Vice-Presidents.*—Dr. W. A. Palmer, Poughkeepsie; Dr. Geo. Allan, Newburgh.

*Secretary.*—L. S. Straw, Newburgh.

*Corresponding Secretary.*—Dr. Dubois, Poughkeepsie.

*Treasurer.*—Dr. C. Royce, Middletown.

*Executive Committee.*—Dr. Shelton, Hudson; Dr. Holley, Warwick; Dr. Finch, Kingston; Dr. Vanduser, Poughkeepsie; Dr. Cross, Rhinebeck.

